# Sex differences in histopathological markers of cerebral amyloid angiopathy and related hemorrhage

**DOI:** 10.1177/17474930241255276

**Published:** 2024-05-24

**Authors:** Emma A Koemans, Valentina Perosa, Whitney M Freeze, Hang Lee, Mariel G Kozberg, Gillian T Coughlan, Rachel F Buckley, Marieke JH Wermer, Steven M Greenberg, Susanne J van Veluw

**Affiliations:** 1 Department of Neurology, Leiden University Medical Center, Leiden, The Netherlands; 2 Department of Neurology, Massachusetts General Hospital, Harvard Medical School, Boston, MA, USA; 3 Department of Biostatistics, Massachusetts General Hospital, Harvard Medical School, Boston, MA, USA; 4 MassGeneral Institute for Neurodegenerative Disease, Massachusetts General Hospital, Harvard Medical School, Boston, MA, USA; 5 Department of Radiology, Leiden University Medical Center, Leiden, The Netherlands

**Keywords:** Cerebral amyloid angiopathy, sex, amyloid, vascular remodeling, intracerebral hemorrhage, histopathology, MRI

## Abstract

**Background:**

Men with cerebral amyloid angiopathy (CAA) may have an earlier onset of intracerebral hemorrhage and a more hemorrhagic disease course compared to women. In this cohort study, we investigated sex differences in histopathological markers associated with amyloid-β burden and hemorrhage in cognitively impaired individuals and patients with CAA, using neuropathological data from two autopsy databases.

**Methods:**

First, we investigated presence of parenchymal (Thal score) and vascular amyloid-β (CAA severity score) in cognitively impaired individuals from the National Alzheimer’s Coordinating Center (NACC) neuropathology database. Next, we examined sex differences in hemorrhagic ex vivo magnetic resonance imaging (MRI) markers and local cortical iron burden and the interaction of sex on factors associated with cortical iron burden (CAA percentage area and vessel remodeling) in patients with pathologically confirmed clinical CAA from the Massachusetts General Hospital (MGH) CAA neuropathology database.

**Results:**

In 6120 individuals from the NACC database (45% women, mean age 80 years), the presence of parenchymal amyloid-β (odds ratio (OR) (95% confidence interval (CI)) =0.68 (0.53–0.88)) but not vascular amyloid-β was less in men compared to women. In 19 patients with definite CAA from the MGH CAA database (35% women, mean age 75 years), a lower microbleed count (p < 0.001) but a higher proportion of cortical superficial siderosis and a higher local cortical iron burden was found in men (p < 0.001) compared to women. CAA percentage area was comparable in men and women (p = 0.732). Exploratory analyses demonstrated a possible stronger negative relation between cortical CAA percentage area and cortical iron density in men compared to women (p = 0.03).

**Conclusion:**

Previously observed sex differences in hemorrhage onset and progression in CAA patients are likely not due to differences in global CAA severity between men and women. Other factors, such as vascular remodeling, may contribute, but future studies are necessary to replicate our findings in larger data sets and to further investigate the underlying mechanisms behind these complex sex differences.

## Introduction

Cerebral amyloid angiopathy (CAA) is one of the leading causes of lobar intracerebral hemorrhage (ICH) and a major contributor to vascular dementia in the elderly.^1^ CAA is characterized by the gradual deposition of the protein amyloid-β in the walls of cortical and leptomeningeal arteries of the brain, which eventually leads to hemorrhage.^2^

Accumulating evidence suggests that hemorrhages occur in the final stages of CAA.^3–5^ Insights from histopathological and pre-clinical studies using mouse models show that amyloid-β initially deposits in the basement membranes surrounding vascular smooth muscle cells. This leads to loss of smooth muscle, thickening of the vessel wall, and eventually vessel wall remodeling.^3,4,6,7^ Two types of vessel wall remodeling have been described: first, Vonsattel grade III vessel remodeling, a concentric splitting of the vessel wall (also called “vessel-within-vessel” appearance) often seen in leptomeningeal arterioles and presumed to be related to cortical superficial siderosis (cSS) and second, Vonsattel grade IV vessel remodeling, characterized by loss of amyloid-β locally with replacement of the arteriolar wall with fibrinoid material, more rarely seen and thought to underly microbleed formation.^3,4,6,8^

Patients with CAA show a striking variability in onset and disease course.^2,9^ This variability is also present in individuals with hereditary Dutch-type CAA (D-CAA), an autosomal dominant variant of CAA which can be considered as a relatively pure, genetic model for the disease.^10^ A previous study from our group suggested that biological sex moderates the presentation of CAA and contributes to variability in onset and disease course. In that study, male sex was associated with an earlier age of first ICH in sporadic CAA, a higher number of ICH recurrences in D-CAA, and a higher microbleed count in sporadic CAA.^11^

We hypothesize that two possible pathophysiological mechanisms may underly the observed sex differences. First, sex may influence global amyloid-β load, resulting in men having earlier or more severe amyloid-β deposition compared to women, which would increase hemorrhage risk or influence age of onset. Second, sex might influence factors leading to bleeding after vascular amyloid-β accumulation (such as vessel remodeling) resulting in men to have a more hemorrhagic CAA phenotype compared to women. With these hypotheses in mind, we explored sex differences in histopathological markers of amyloid-β and hemorrhage in CAA using two different autopsy cohorts.

### Study aims

The overall aim of our study was to investigate possible pathophysiological mechanisms for sex associated with CAA disease course. First, we aimed to investigate sex differences in presence of vascular and parenchymal amyloid-β deposition in cognitively impaired autopsied individuals. To investigate this, we used clinical and neuropathological data from the National Alzheimer’s Coordinating Center (NACC) database.

Second, we aimed to investigate sex differences in factors associated with hemorrhage at advanced stages of CAA disease progression. To investigate this, we used ex vivo magnetic resonance imaging (MRI) and artificial intelligence (AI)-derived quantitative neuropathological data from patients with a pathologically confirmed clinical diagnosis of CAA from the Massachusetts General Hospital (MGH) CAA database.

## Methods

This autopsy cohort study was reported according to the STrengthening the Reporting of OBservational studies in Epidemiology (STROBE) guidelines.

### Data sharing statement

Further information about the data sets is available from the corresponding author upon reasonable request.

### NACC neuropathology database

We investigated our first hypothesis using data from the NACC neuropathology database. The NACC neuropathology database contains longitudinal clinical and neuropathological data from participants collected via National Institute on Aging funded Alzheimer’s Disease Research Centers (ADRCs) across the United States since 2005, with different underlying etiologies including Alzheimer’s disease and Parkinson’s disease as well as healthy individuals.^12^ Our current study includes individuals recruited within the period from 2005 until April 2022. Informed consent was obtained from all participants or next of kin prior to study enrollment, and the study was approved by the local institutional review boards of each participating institution. The database contains coded demographics, clinical data (including physician diagnosis of hypertension and hypercholesterolemia), genetic data (including *APOE-*ε4 status, a known risk factor for CAA and Alzheimer’s disease), and neuropathology data obtained after autopsy (including CAA severity score for vascular amyloid-β and Thal score for parenchymal amyloid-β).^12^ All participants in the NACC database underwent cognitive testing, which took place during each research visit. The database includes participants with normal cognitive function, as well as three stages of cognitive impairment: subjective impaired cognition, mild cognitive impairment (MCI), and dementia (for further details on the NACC neuropathology database, see Supplemental methods).

### MGH CAA neuropathology database

We investigated our second hypothesis using data from patients with a pathologically confirmed clinical diagnosis of CAA, collected at MGH. The MGH CAA neuropathology database, initiated in 2015 within the hemorrhagic stroke research program, aims to study the neuropathological correlates of MRI manifestations of CAA. The study has been approved by the MGH institutional review board (2021P001920), and informed consent was obtained prior to autopsy from brain donors’ next of kin or other legal representatives. Details on the autopsy procedure and data collection has been described previously.^8,10,11^ To summarize: after autopsy, one formalin-fixed hemisphere underwent routine neuropathological examination by a board-certified neuropathologist, whereas the other (most intact) formalin-fixed hemisphere was subjected to ex vivo 3 tesla MRI and histopathological examination. The single-hemisphere ex vivo MRI scans were visually assessed for CAA-related MRI markers (macrobleeds, microbleeds, cSS), defined according to the Standards for Reporting Vascular Changes on Neuroimaging (STRIVE) criteria, as previously reported.^3,4,13,14^

**Figure 1.**
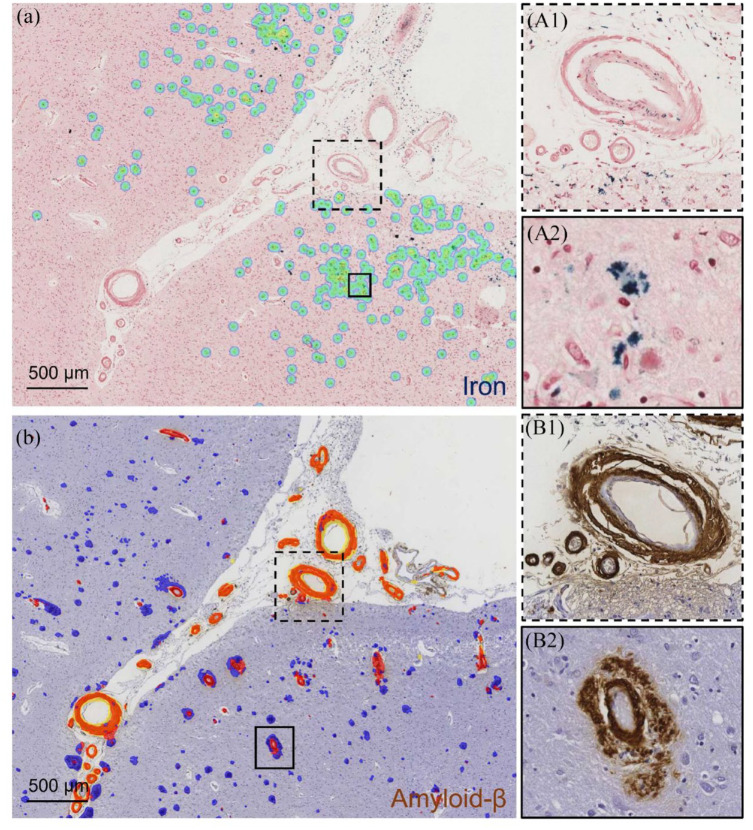
Examples of iron deposits in the cortex, cortical and leptomeningeal CAA and leptomeningeal grade III vessel remodeling. Examples of the application of a deep learning model (Aiforia®) for iron (a) and amyloid (b) in two adjacent brain sections of a participant with CAA. (a) Cortical iron deposits recognized by the model are overlaid in green (stained blue, see corresponding inset A2). (b) Parenchymal amyloid-β recognized by the model is overlaid in blue, CAA (cortical and leptomeningeal) recognized by the model in red (see corresponding inset B1 and B2, amyloid-β stained brown). Inset A1 and B1 shows an example of a single leptomeningeal vessel with Vonsattel grade III remodeling (vessel-in-vessel pathology). The inset B2 shows an example of a cortical vessel with dyshoric CAA.

Using data from the MGH neuropathology database, first, we investigated sex differences in hemorrhagic MRI markers (macrobleeds, microbleeds, and cSS) on ex vivo 3 tesla MRI between men and women. Second, we investigated sex differences in density of cortical iron deposition, a quantitative measure derived from deep learning-based models (see Figure 1),^15^ which was operationally defined as a proxy for cumulative local hemorrhage severity.^16^ Although cortical iron density can be considered a measure of all hemorrhagic lesions (microbleeds, macrobleeds, and cSS), our work suggests that iron density predominantly reflects cSS severity.^3,15,16^ Finally, we explored sex differences in CAA by taking into account previously investigated factors associated with hemorrhage (and therefore likely contributing to cortical iron deposition): cortical and leptomeningeal CAA area percentage and degree of leptomeningeal vessel remodeling (Vonsattel grade III, see Figure 1^3,6,8^ or further details on the MGH CAA neuropathology database and the specific tissue analyses, see Supplemental methods).

### Statistics

#### NACC neuropathology database

Descriptive statistics were utilized to summarize the distributions of the baseline characteristics. To investigate sex differences in vascular amyloid-β (CAA severity score) and parenchymal amyloid-β (Thal phase), we applied generalized estimating equation (GEE) for logistic regression with a logit link and independent correlation structure specification, all corrected for age at death. Because of a relatively low sample size of participants with CAA, we transformed the categorical global CAA burden score into a binary variable: absent versus present (CAA severity mild/moderate/severe). In sensitivity analysis, we repeated the analysis using a modified variable (absent-mild CAA vs moderate-severe CAA), and by transforming Thal phase into a binary variable (absent (A0) vs present (A1/A2/A3)). Given the significant contribution of age and *APOE*-ε4 status (dichotomized as presence of at least 1 ε4 allele vs none) on parenchymal amyloid-β burden, we corrected these in all models. In the model using CAA as a dependent variable, we additionally corrected for Thal phase to investigate a possible confounding effect (for details regarding the statistical analyses and the post hoc analyses, see Supplemental methods).

#### MGH CAA neuropathology database

Descriptive statistics were utilized to summarize the distributions of the baseline characteristics. First, we investigated sex differences in hemorrhagic lesions at ex vivo single-hemisphere 3 tesla MRI. We investigated sex differences in macro- and micro-bleed count and cSS frequency using the Poisson regression analysis, corrected for age at death, to obtain adjusted relative risks (aRRs) with 95% confidence interval (CI). Second, we investigated sex differences in total CAA percentage area, cortical iron density (as a measure for hemorrhage load but mostly reflecting cSS), again using the Poisson regression analysis corrected for age at death. To examine the association between male sex and cortical iron density, we fit linear mixed effect (LME) models using the package “lme4” in R. All models contained density of iron positive deposits/mm^2^ cortex as a dependent variable, and subject ID and cortical region as random factors to account for subject- and region-dependent differences. Age at death was included as covariate in the model. First, we fitted the null model which included age at death and sex. Second, we fitted three sets of models: a first model with leptomeningeal grade III vessel remodeling and sex as interaction factor, a second model with leptomeningeal CAA area and sex as interaction factor, and a third model with cortical CAA area and sex as interaction factor (Table 4). Models were compared using the likelihood ratio tests, and analysis of variance (ANOVA) tests were used to compare the individual models to the null model. Finally, we fitted a comprehensive model including all independent variables which showed a significant association with cortical iron density in prior analyses. We plotted scatterplots to illustrate the relation between leptomeningeal grade III vessel remodeling and iron density, and cortical CAA area percentage and iron density, stratified by sex, and visually checked for outliers. A two-sided p < 0.05 was considered statistically significant.

## Results

### NACC neuropathology database

We included 6120 participants with autopsy data from the NACC database, 3370 (55%) men and 2750 (45%) women, mean age at death 81 years (range = 50–111) (Table 1). Men more often used antiplatelet therapy or anticoagulants (42% vs 35%). The GEE analysis investigating sex differences in presence of vascular amyloid-β (CAA) consisted of 2844 individuals. Presence of vascular amyloid-β indicative of CAA did not differ between men and women (odds ratio (OR) (95% CI) = 1.05 (0.87–1.27)), correcting for age, *APOE*, and Thal stage. The GEE analysis investigating sex differences in the presence of parenchymal amyloid-β (Thal phase) consisted of 2854 individuals. Men were less likely to have parenchymal amyloid-β in the cortex (i.e. Thal score > A0) compared to women (OR (95% CI) = 0.68 (0.53–0.87)). The outcomes did not change when using ordinal regression investigating sex differences in CAA severity score or Thal score (as categorical variables) instead of logistic regression nor did the outcomes change when investigating absent-mild CAA versus moderate-severe (Table 2). Correction for history of hypercholesterolemia and hypertension also did not change the outcomes (Table 2).

**Table 1.** Baseline characteristics of the NACC neuropathology database from participants with available neuropathology data.

	All (n = 6120)	Men (n = 3370)	Women (n = 2750)
Mean age at death in years (range)	80 (50-111)	78 (50-110)	81 (50-111)
Use of anticoagulants or antiplatelet therapy (%)^a^	2347 (38)	1399 (42)	948 (35)
Mean interval between last UDS visit and death in months (range)	20 (0-161)	18 (0-152)	22 (0-161)
*APOE*-ε4^b^			
One ε4 allele (n, %)	2048 (34)	1114 (33)	934 (34)
Two ε4 alleles (n, %)	504 (8)	285 (9)	219 (8)
Hypertension (n, %)^c^	848 (14)	459 (14)	389 (14)
Hypercholesterolemia (n, %)^d^	805 (13)	469 (14)	336 (12)
CAA pathology at autopsy^e^			
None (n, %)	2266 (37)	1278 (38)	988 (36)
Mild (n, %)	1722 (28)	926 (28)	796 (29)
Moderate (n, %)	1295 (21)	697 (21)	598 (22)
Severe (n, %)	707 (12)	402 (12)	305 (11)
Thal phase at autopsy^f^			
A0 (n, %)	384 (6)	245 (7)	139 (5)
A1 (n, %)	410 (7)	237 (7)	173 (6)
A2 (n, %)	360 (6)	188 (6)	172 (6)
A3 (n, %)	2125 (35)	1104 (33)	1021 (37)

a Missing/unknown n = 203.

b Missing/unknown n = 737.

c Missing/unknown n = 4385.

d Missing/unknown n = 4414.

e Missing/unknown n = 130.

f Missing/unknown n = 2841.

**Table 2.** Estimated odds ratios from the NACC neuropathology database.

	Vascular amyloid-β (CAA severity score) (n = 2844)	Parenchymal amyloid-β (Thal score) (n = 2854)
	OR [95% CI]	OR [95% CI]
Male sex^a,b^	1.052 [0.872–1.269]^c,d^	0.680 [0.528–0.876]*^e^
Age at death	1.003 [0.995–1.012]	1.040 [1.028–1.052]*
*APOE*-ε4 status positive	2.505 [2.065–3.039]*	12.68 [8.512–18.890]*
Thal phase A1	5.958 [3.677–9.655]*	–
Thal phase A2	13.837 [8.395–22.811]*	–
Thal phase A3	37.764 [24.083–59.218]*	–

B: raw parameter estimate (on logit scale); SE: robust (sandwich) standard error; OR: odds ratio; 95% CI: 95% confidence interval.

a Modifying the analysis to compare CAA stage non-mild versus moderate-severe did not change the model outcomes (effect male sex on CAA: OR (95% CI) = 1.049 (0.887–1.241)).

b Ordinal regression with CAA severity score and Thal phase as ordinal dependent variables using GEE did not change the model outcomes (effect male sex on CAA: OR (95% CI) = 1.080 (0.911–1.280) and effect male sex on Thal phase: OR (95% CI) = 0.810 (0.701–0.937)).

c Running the model without including Thal stage as a factor did not change the model outcomes (effect male sex on CAA: OR (95% CI) = 1.000 (0.887–1.127)).

d Adding possible confounders hypertension and hypercholesterolemia to the model did not change the model outcome but did drastically decrease the sample size (OR (95% CI) = 1.118 (0.867–1.441), n = 1481).

e Adding possible confounders hypertension and hypercholesterolemia to the model did not change the model outcome but did drastically decrease the sample size (OR (95% CI) = 0.679 (0.479–0.962), n = 1487).

* Statistically significant (p < 0.05).

### MGH CAA neuropathology database

We included 19 patients with definite CAA (mean age at death 75 years, 7 (35%) women) (Table 3). Men had lower microbleed counts on ex vivo MRI compared to women (median 45 vs 109; aRR = 0.39, p < 0.001), whereas men numerically had more often cSS on ex vivo MRI compared to women (67% vs 29%, aRR = −0.91, p = 0.25). On neuropathology, men had a higher local cortical iron density compared to women (median 6.42/mm^2^ vs 5.13/mm^2^, aRR = −0.75, p < 0.001), which is consistent with an overall greater cSS burden in men. Total CAA percentage area was comparable between men and women (median 1.32 vs 1.20; aRR = −0.15, p = 0.73).

**Table 3.** Baseline characteristics of the MGH CAA neuropathology database.

	Total (n = 19)	Men (n = 12)	Women (n = 7)	aRR (95% CI) [p-value]
Mean age at death in years (range)	75 (65-89)	74 (65-86)	76 (65-89)	–
Ex vivo 3 tesla MRI (single hemisphere)				
Macrobleed prevalence (%)	11 (58)	6 (50)	5 (71)	–
Median macrobleed count (range)	1 (0-5)	0.5 (0-5)	2 (0-3)	0.44 (-0.35, 1.22) [0.261]
Microbleed prevalence (%)	10 (100)	12 (100)	7 (100)	–
Median microbleed count (range)	49 (4-261)	45 (4-261)	109 (9-204)	**0.39 (0.29, 0.49) [<0.001]**
cSS (n, %)	10 (53)	8 (67)	2 (29)	-0.91 (-2.8, 0.49) [0.253]
Histopathology				
Median count of iron deposits/mm^2^ in cortex (range)^a^	5.34 (0.29-71.80)	6.42 (0.73-71.80)	5.13 (0.27-14.78)	**-0.75 (-1.11, -0.41) [<0.001]**
Median Total CAA area (%, range)^a^	1.26 (0.40-2.71)	1.32 (0.40-2.71)	1.20 (0.72-1.63)	-0.15 (-1.04, -0.67) [0.732]
Median Cortical CAA area (%, range)^a^	0.78 (0.26-1.44)	1.01 (0.26-1.44)	0.75 (0.39-1.12)	–
Median Leptomeningeal CAA area (%, range)^a^	28.30 (8.89-57.90)	19.88 (8.88-57.90)	41.11 (15.69-46.09)	–
Median leptomeningeal vessel remodeling score^b^ (range)	4 (0-8)	5 (0-8)	3 (1-7)	–

a All scores for whole brain obtained by adding regional scores and dividing by number of regions used.

b Scores per region added to create a score for whole brain between 0 and 8.

Significant effects are highlighted in bold.

Next, we examined the relation between sex and cortical iron density in further detail using LME models. The model included all 19 cases, together a total of 71 sections (5 sections were excluded because of inaccurate detections by the Aiforia algorithm). Results are summarized in Table 4. The variables that showed a significant main association with cortical iron density were leptomeningeal vessel remodeling, in model 1 and 4 (a positive association) and cortical CAA area percentage, in model 4 (a negative association).

**Table 4.** Estimated sex differences in factors influencing cortical iron density.

	(0) M0	(1a) with leptomeningeal vessel remodeling	(1b) with leptomeningeal vessel remodeling interaction with sex	(2a) with leptomeningeal CAA area (%)	(2b) with leptomeningeal CAA area (%): sex	(3a) with cortical CAA area (%)	(3b) with cortical CAA area (%): sex	(4) comprehensive model
Age at death (years)	estimate = –0.541 (–1.412–0.432)^a^ p = 0.231	estimate = –0.6241 (–1.468–0.218) p = 0.143	estimate = –0.649 (–1.458–0.159) p = 0.114	estimate = –0.640 (–1.525–0.255) p = 0.154	estimate = –0.740 (–1.596–0.135) p = 0.094	estimate = –0.577 (–13.108–3.392) p = 0.182	estimate = –0.522 (–1.331–0.2888) p = 0.199	estimate = –0.613 (–1.368–0.144) p = 0.111
Sex (men 0, women 1)	estimate = –5.252 (–2.080–0.886)^a^ p = 0.473	estimate = –3.147 (–17.012–10.728) p = 0.646	estimate = 4.437 (–12.391–20.779) p = 0.592	estimate = –5.667 (–19.876–8.667) p = 0.423	estimate = 9.272 (–19.172–36.698) p = 0.505	Estimate = –5.687 (–19.679–8.476) p = 0.415	**estimate** **=** **–18.958 (–36.549 to –0.832)** **p** **=** **0.038**	estimate = –14.236 (–31.180–3.315) p = 0.102
Leptomeningeal grade III vessel remodeling		**estimate** **=** **7.189 (2.324–12.059)** **p** **=** **0.005**	**estimate** **=** **9.735 (3.853–15.618)** **p** **=** **0.002**					**estimate** **=** **6.545 (1.749–11.335**) **p** **=** **0.008**
Leptomeningeal grade III vessel remodeling: sex			estimate = –7.686 (–18.034–2.609) p = 0.139					
Leptomeningeal CAA area (%)				estimate = 0.170 (–0.164–0.492) p = 0.299	estimate = 0.303 (–0.091–0.676) p = 0.115			
Leptomeningeal CAA area (%): sex					estimate = –0.434 (–1.133–0.287) p = 0.222			
Cortical CAA area (%)						estimate = –4.733 (–13.108–3.392) p = 0.235	**estimate** **=** **–11.352 (–21.378 to –1.384)** **p** **=** **0.022**	**estimate** **=** **–10.362 (–20.040 to –0.752)** **p** **=** **0.028**
Cortical CAA area (%): sex							**estimate** **=** **17.191 (1.351–33.279)** **p** **=** **0.034**	*estimate* *=* *13.516 (–1.97–29.276)* *p* *=* *0.084*
Log likelihood	–284.0	–279.9	–278.9	–283.5	–282.8	–283.4	–281	–278
AIC	580	574	573	581	582	580	578	573
BIC	593	589	591	597	599	596	596	593
ANOVA outcome (m0, . . .)		**0.004364**	0.141242	0.3066	0.2307	0.2521	**0.03377**	**0.005082**

Results are obtained from the fixed effects of linear mixed effects (LME) models looking at the influence of sex on cortical iron density. Subject and cortical region (frontal, temporal, parietal, occipital) were set as random factors for the intercept. Data represent standardized fixed effects estimates with confidence intervals and statistical significance. Models were compared using likelihood ratio tests; smaller AIC and BIC values indicate a better model fit.

a 95% CI obtained via bootstrapping.

Significant effects are highlighted in bold; trend toward significance are highlighted in italics.

To visualize our data, we plotted scatterplots for the relation between cortical CAA area percentage and cortical iron density, and leptomeningeal grade III vessel remodeling and cortical iron density, for men and women (Supplemental Figure 1). The scatterplots suggested an opposite relation of cortical CAA area percentage with cortical iron density in men and women (Supplemental Figure 2A and Supplemental Table 1). This interaction effect of sex was statistically significant (p = 0.034, model 3b), suggesting sex moderates this relationship. This significance became attenuated in a more comprehensive model 4 (p = 0.08). Leptomeningeal CAA area percentage was not associated with cortical iron density, nor was there an interaction effect of sex.

## Discussion

In this study, we explored sex differences in CAA histopathology. In an autopsy cohort of cognitively impaired individuals, women exhibited a higher prevalence of parenchymal amyloid-β (Thal staging) compared to men. In contrast, no sex differences were observed in the presence of vascular amyloid-β (CAA). In a different cohort of patients with neuropathologically confirmed CAA, we found significantly more microbleeds on ex vivo MRI in women, but a significantly higher local cortical iron burden—in line with greater burden of cSS—in men. Total CAA percentage area was comparable between men and women in this database. Exploratory analyses suggested that the strongest contributing factor to local cortical iron burden was leptomeningeal grade III vessel remodeling. Furthermore, we found that higher cortical CAA area percentage was related to a lower cortical iron density, and that sex moderated this association, such that the association was stronger in men compared to women.

The finding that female sex is related to higher parenchymal amyloid-β prevalence is consistent with previous studies, which found that women exhibit elevated Alzheimer’s disease pathology compared to age-matched men.^17,18^ Our results were inconsistent with a similar autopsy study performed in participants with Alzheimer’s disease, which demonstrated that men have higher vascular CAA severity scores compared to women.^19^ This discrepancy might be due to a difference in CAA severity categorization between this study and the NACC database. Furthermore, the NACC database contains a larger but more heterogeneous population, whereas the previous study used autopsy data of individuals with neuropathologically confirmed Alzheimer’s disease. Based on our current observations, it seems unlikely that the previously identified differences in ICH onset and recurrence between men and women with CAA can sufficiently be explained by differences in global CAA severity between both sexes.^11^

On ex vivo MRI, we found higher microbleed counts in women compared to men. This is inconsistent with our previous in vivo study that demonstrated that men had a higher prevalence and count of microbleeds. Similarly, these ex vivo results contradict a previous in vivo study in Alzheimer’s disease which also found higher lobar microbleed counts in men compared to women.^11,20^ We speculate these discrepancies might be due to the differences in sample size, as well as the differences in disease stage (the MGH CAA neuropathology database being an end-stage cohort by design).

We next explored sex differences in cortical iron burden, which was higher in men compared to women. Since local iron burden as measured here predominantly reflects cSS, this effect is likely driven by the fact that men in this cohort more often had cSS compared to women.^3,15^

We furthermore explored the possible interaction effect of sex on factors contributing to cortical iron burden. We observed a negative association of local cortical CAA area percentage with local cortical iron density. This is in line with previous observations in the same cohort, where we found a strong relationship between increased leptomeningeal CAA severity and cSS in the form of local iron burden, and a negative association between cortical CAA severity and local iron burden.^3^ It is intriguing that our data (Supplemental Figure 1A) suggest that this negative association is more apparent in men than in women with CAA, although men had fewer microbleeds compared to women. This again may be because in the men of our population, cSS is probably the major contributing factor to local cortical iron burden, and cSS is not associated with cortical CAA.^3^ The previously observed relationship between leptomeningeal CAA area percentage and cortical iron burden could not be confirmed, possibly because of the inclusion of additional cases between the prior study and now.^3^ Future studies are warranted to address the relationship between sex and neuroinflammation, which has been implicated in the pathophysiology of vessel remodeling and hemorrhage in CAA. Moreover, it remains unknown whether sex differences exist in the clinical condition of CAA-related inflammation, another area for further research.^8,21^

This study has several limitations. First, the NACC data collection is not population-based. Therefore, the data are heterogeneous and subject to enrollment bias. Furthermore, the NACC database did not include information on hemorrhagic lesions and amyloid-β presence was scored solely on a visual, categorical scale, a less specific variable compared to the AI-derived continuous measures used in the MGH CAA neuropathology database. It is possible that there are substantial differences in local CAA severity that was not detected because of the used methods. Although the NACC database is very extensive, many patients have missing data and, therefore, we had to exclude them from some of our analyses. Men in the NACC database more often used anticoagulants/antiplatelet therapy; however, we do not expect this to have influenced the investigated outcomes. Second, as mentioned above, the sample size in the MGH CAA neuropathology database was small. Moreover, it is a selective cohort, because by study design, the less affected hemisphere is designated for ex vivo MRI and histopathological analysis. This may have led to a relative under-representation of hemorrhagic MRI lesions. Via the statistical methods used in this article, we aimed to correct for random effects. Despite this, we consider our study mainly exploratory, and our results need to be interpreted with care and should be replicated in larger cohorts. Third, the use of autopsy data limits the observations to the final disease stage. Therefore, any sex differences which could have been present during life may no longer be seen in these data.^11^ Furthermore, we did not have information regarding cause of death for all patients in the MGH CAA neuropathology database. Finally, by focusing solely on leptomeningeal grade III vessel remodeling, our methods may be more relevant in uncovering sex differences in cSS and ICH pathophysiology, whereas the implications for microbleed pathophysiology warrants further investigation. Future studies are warranted to investigate the effect of sex on grade IV vessel remodeling, although this is challenging as these vessels are rarely observed on routine neuropathological examination.^8^

The results of this exploratory study suggest that, although sex may influence (parenchymal) amyloid-β accumulation and (factors contributing to) hemorrhage burden in CAA, the mechanisms behind these differences are complex and should be investigated in more detail in larger CAA cohorts. It is important to consider that the mechanisms might depend on (epi) genetic or environmental factors, or sex-specific hormones such as estrogen and testosterone. Future studies need to investigate sex-specific mechanisms that underlie the observed differences, as these could be possible targets for treatment or prevention in CAA.

## Supplemental Material

sj-docx-1-wso-10.1177_17474930241255276 – Supplemental material for Sex differences in histopathological markers of cerebral amyloid angiopathy and related hemorrhage Supplemental material, sj-docx-1-wso-10.1177_17474930241255276 for Sex differences in histopathological markers of cerebral amyloid angiopathy and related hemorrhage by Emma A Koemans, Valentina Perosa, Whitney M Freeze, Hang Lee, Mariel G Kozberg, Gillian T Coughlan, Rachel F Buckley, Marieke JH Wermer, Steven M Greenberg and Susanne J van Veluw in International Journal of Stroke
