# Supplementary material for: Sex differences in histopathological markers of cerebral amyloid angiopathy and related hemorrhage
Source: Int J Stroke. 2024 May 24;19(8):947–56. doi: 10.1177/17474930241255276 (PMC11408965; doi:10.1177/17474930241255276)
Supplement: sj-docx-1-wso-10.1177_17474930241255276 – Supplemental material for Sex differences in histopathological markers of cerebral amyloid angiopathy and related hemorrhage [file sj-docx-1-wso-10.1177_17474930241255276.docx]

**Supplemental material**

Supplemental methods

***Histopathology in the NACC neuropathology database***

In the NACC neuropathology database, immunohistochemistry against amyloid-β was applied using different antibodies. Vascular amyloid-β indicating CAA severity was measured using a semi-quantitative scale adapted from prior studies.(2, 3) CAA burden was described as 0 = absent, 1 = mild (scattered positivity in parenchymal and/or leptomeningeal vessels), 2 = moderate (intense positivity in many parenchymal and/or leptomeningeal vessels), 3 = severe (widespread [more than one brain area] intensive positivity in parenchymal and leptomeningeal vessels).(2) Parenchymal amyloid-β (plaque burden) was scored according to Thal score, a previously published system which distinguishes 4 phases of amyloid-β plaque severity according to anatomical progression: A0 = no amyloid-β; A1 = isocortical amyloid-β and/or limbic amyloid-β; A2 = basal ganglia amyloid-β; A3 = basal forebrain and midbrain amyloid-β, and/or pons/medulla oblongata and cerebellum amyloid-β.(4, 5)

***Histopathology in the MGH CAA neuropathology database***
Details regarding the preparation of the histopathologic samples can be found in previous publications from our group.(7, 8, 10) To summarize: samples from pre-defined areas of the brain were cut from 1cm thick coronal slabs (frontal, temporal, parietal, and occipital). Blocks were processed and embedded in paraffin, after which 6-μm thin sections were cut using a microtome. Haematoxylin and eosin (H&E) and Perls’ Prussian blue staining were performed according to standard histology protocols. For the Perls’ protocol, sections were incubated using a 1:1 mixture of 5% hydrochloric acid and 5% potassium ferrocyanide for 30 minutes, and afterwards counterstained with filtered neutral red for 1 min.(7) Immunohistochemistry was performed against amyloid-β as previously described on adjacent sections.(7, 8, 10) The stained sections were scanned using a Hamamatsu NanoZoomer Digital Pathology (NDP)-HT whole slide scanner (C9600-12, Hamamatsu Photonics KK, Japan), at 20 × magnification.

The following parameters were calculated: percentage of leptomeningeal CAA area (leptomeningeal CAA area [mm^2^]/leptomeningeal tissue area [mm^2^]); percentage of cortical CAA area (cortical CAA area [mm^2^]/cortical tissue area [mm^2^]); density of iron positive deposits/mm^2^ cortex (Total count of iron deposits/cortex area [mm^2^]) (Figure 1).(10) Calculations were performed separately for each individual brain region (frontal, parietal, temporal, occipital). For descriptive statistics, mean quantitative scores for the whole brain were obtained in the following way: added total scores of all brain regions/number of brain regions. We used existing data in which the degree of leptomeningeal grade III vessel remodelling was quantified. For each brain region, the degree of leptomeningeal grade III vessel remodelling was determined according to the following score: 0 = absent, 1 = occasional vessel, 2 = many vessels (Figure 1).(7) For descriptive statistics, a total leptomeningeal grade III vessel remodelling score was calculated for each individual by adding the scores of the four brain regions to create a score between 0-8. We investigated the leptomeningeal grade III vessel remodelling as previous studies have shown that it is strongly associated with blood-brain barrier leakage and hemorrhage (cSS and ICH) in CAA.(2, 6-8, 11) Unfortunately, we could not investigate the effect of (parenchymal) grade IV vessel remodelling: whereas grade III vessel remodelling was often seen and could be categorised in leptomeningeal tissue of our participants with CAA, grade IV vessel remodelling was seen very rarely, and therefore, the counts of grade IV vessel remodelling in our cohort were too low to be included in the analyses.(6-8)

**Statistics**

*NACC neuropathology database*

We performed statistical analysis using the software R studio, version 3.6.0 (R Foundation for Statistical Computing, Vienna, Austria; www.R-project.org) and the Statistical Package for Social Science (IBM SPSS Statistics), version 26. We used package ‘gee’ for R statistical software to fit generalized linear models (GLM) using generalized estimating equations (GEE) approach. In exploratory post-hoc analyses, we fitted a second model for both outcomes, in which we additionally corrected for presence of cardiovascular risk factors (hypertension and hypercholesterolemia). Furthermore, we examined sex differences in CAA severity and Thal phase using the outcomes as ordinal variables (four ordinal levels). We utilized a cumulative logit link function for ordinal outcome, again with an independent correlation structure to account for within-center correlation. A two-sided p<0.05 was considered statistically significant. In case of missing values participants were excluded from the specific analyses.

**Acknowledgements**This work was conducted with support from Harvard Catalyst | The Harvard Clinical and Translational Science Center (National Center for Advancing Translational Sciences, National Institutes of Health Award UL1 TR002541) and financial contributions from Harvard University and its affiliated academic healthcare centers. The content is solely the responsibility of the authors and does not necessarily represent the official views of Harvard Catalyst, Harvard University and its affiliated academic healthcare centers, or the National Institutes of Health.
The NACC database is funded by NIA/NIH Grant U24 AG072122. NACC data are contributed by the NIA-funded ADRCs: P30 AG062429 (PI James Brewer, MD, PhD), P30 AG066468 (PI Oscar Lopez, MD), P30 AG062421 (PI Bradley Hyman, MD, PhD), P30 AG066509 (PI Thomas Grabowski, MD), P30 AG066514 (PI Mary Sano, PhD), P30 AG066530 (PI Helena Chui, MD), P30 AG066507 (PI Marilyn Albert, PhD), P30 AG066444 (PI John Morris, MD), P30 AG066518 (PI Jeffrey Kaye, MD), P30 AG066512 (PI Thomas Wisniewski, MD), P30 AG066462 (PI Scott Small, MD), P30 AG072979 (PI David Wolk, MD), P30 AG072972 (PI Charles DeCarli, MD), P30 AG072976 (PI Andrew Saykin, PsyD), P30 AG072975 (PI David Bennett, MD), P30 AG072978 (PI Neil Kowall, MD), P30 AG072977 (PI Robert Vassar, PhD), P30 AG066519 (PI Frank LaFerla, PhD), P30 AG062677 (PI Ronald Petersen, MD, PhD), P30 AG079280 (PI Eric Reiman, MD), P30 AG062422 (PI Gil Rabinovici, MD), P30 AG066511 (PI Allan Levey, MD, PhD), P30 AG072946 (PI Linda Van Eldik, PhD), P30 AG062715 (PI Sanjay Asthana, MD, FRCP), P30 AG072973 (PI Russell Swerdlow, MD), P30 AG066506 (PI Todd Golde, MD, PhD), P30 AG066508 (PI Stephen Strittmatter, MD, PhD), P30 AG066515 (PI Victor Henderson, MD, MS), P30 AG072947 (PI Suzanne Craft, PhD), P30 AG072931 (PI Henry Paulson, MD, PhD), P30 AG066546 (PI Sudha Seshadri, MD), P20 AG068024 (PI Erik Roberson, MD, PhD), P20 AG068053 (PI Justin Miller, PhD), P20 AG068077 (PI Gary Rosenberg, MD), P20 AG068082 (PI Angela Jefferson, PhD), P30 AG072958 (PI Heather Whitson, MD), P30 AG072959 (PI James Leverenz, MD).

Supplemental results

**Supplemental Table 1**: Estimated sex differences in factors influencing cortical iron density, including outcomes without outlier.

|  | **(0) M0** | **(1a) with leptomeningeal vessel remodelling** | **(1b) with leptomeningeal vessel remodelling interaction with sex** | **(2a) with leptomeningeal CAA area (%)** | **(2b) with leptomeningeal CAA area (%) : sex** | **(3a) with cortical CAA area (%)** | **(3b) with cortical CAA area (%) : sex** | **(4) comprehensive model** |
| --- | --- | --- | --- | --- | --- | --- | --- | --- |
| Age at death (y) | estimate= -0.541  (-1.412-0.432)^1^ p=0.231  Without outlier: estimate: -0.43, p=0.19 | estimate= -0.6241  (-1.468-0.218) p=0.143  Without outlier: estimate: -0.52, p=0.10 | estimate= -0.649 (-1.458-0.159) p=0.114  Without outlier: *estimate: -0.54, p=0.08* | estimate= -0.640 (-1.525-0.255) p=0.154  Without outlier: estimate: -0.49, p=0.14 | estimate= -0.740 (-1.596-0.135) p=0.094  Without outlier: *estimate: -0.57, p=0.08* | estimate= -0.577  (-13.108-3.392) p=0.182  Without outlier: estimate: -0.46, p=0.27 | estimate= -0.522 (-1.331-0.2888) p=0.199  Without outlier: estimate: -0.41, p=0.16 | estimate= -0.613 (-1.368-0.144) p=0.111  Without outlier: *estimate: -0.5, p=0.08* |
| Sex (male 0, female 1) | estimate= -5.252  (-2.080-0.886)^1^ p=0.473  Without outlier: estimate: -3.8, p=0.47 | estimate= -3.147  (-17.012-10.728) p=0.646  Without outlier: estimate: -1.89, p=0.71 | estimate= 4.437  (-12.391-20.779) p=0.592  Without outlier: estimate: 4.67 p=0.43 | estimate= -5.667 (-19.876-8.667) p=0.423  Without outlier: estimate: -4.04, p=0.44 | estimate= 9.272  (-19.172-36.698) p=0.505  Without outlier: estimate: 7.15, p=0.48 | estimate= -5.687  (-19.679-8.476) Pp0.415  Without outlier: estimate: -4.10, p=0.42 | **estimate= -18.958 (-36.549- -0.832) p=0.038**  Without outlier: **estimate: -15.68, p=0.02** | estimate= -14.236 (-31.180-3.315) p=0.102  Without outlier: *estimate: -11.12, p=0.07* |
| Leptomeningeal grade III vessel remodelling |  | **estimate= 7.189 (2.324-12.059) p=0.005**  Without outlier: **estimate: 6.63, p=0.00** | **estimate= 9.735 (3.853-15.618) p=0.002**  Without outlier: **estimate: 8.81 p<0.01** |  |  |  |  | **estimate= 6.545 (1.749-11.335**) **p0.008**  Without outlier: **estimate: 5.97, p<0.01** |
| Leptomeningeal grade III vessel remodelling :  sex |  |  | estimate= -7.686 (-18.034-2.609) p=0.139  Without outlier: *estimate: -6.66 p=0.07* |  |  |  |  |  |
| Leptomeningeal CAA area (%) |  |  |  | estimate= 0.170  (-0.164-0.492) p=0.299  Without outlier: estimate: 0.1, p=0.42 | estimate= 0.303  (-0.091-0.676) p=0.115  Without outlier: estimate: 0.2, p=0.16 |  |  |  |
| Leptomeningeal CAA area (%) : sex |  |  |  |  | estimate= -0.434 (-1.133-0.287) p=0.222  Without outlier: estimate: -0.32, p=0.21 |  |  |  |
| Cortical CAA area (%) |  |  |  |  |  | estimate= -4.733  (-13.108-3.392) p=0.235  Without outlier: estimate: -3.25, p-0.27 | **estimate= -11.352 ( -21.378- -1.384) p=0.022**  Without outlier: **estimate: -9.00, p=0.01** | **estimate= -10.362 (-20.040- -0.752) p=0.028**  Without outlier: **estimate: -7.76, p=0.02** |
| Cortical CAA area (%) : sex |  |  |  |  |  |  | **estimate= 17.191 (1.351-33.279) p=0.034**  Without outlier: **estimate: 15.68, p=0.01** | *estimate= 13.516 (-1.97-29.276) p=0.084*  Without outlier: **estimate: 11.31 p=0.04** |
|  |  |  |  |  |  |  |  |  |
| Log likelihood | -284.0 | -279.9 | -278.9 | -283.5 | -282.8 | -283.4 | -281 | -278 |
| AIC | 580 | 574 | 573 | 581 | 582 | 580 | 578 | 573 |
| BIC | 593 | 589 | 591 | 597 | 599 | 596 | 596 | 593 |
| Anova outcome (m0,…) |  | **0.004364**  Without outlier **0.000258** | 0.141242  Without outlier *0.067600* | 0.3066  Without outlier 0.4223 | 0.2307  Without outlier 0.2328 | 0.2521  Without outlier 0.2941 | **0.03377**  Without outlier **0.01132** | **0.005082**  Without outlier **0.0002784** |

Results were from the fixed effects of linear mixed effects (LME) models looking at the influence of sex on cortical iron density. Subject and cortical region (frontal, temporal, parietal, occipital) were set as random factors for the intercept. Data represent standardized fixed effects estimates with confidence intervals and statistical significance. Models were compared using likelihood ratio tests; smaller AIC and BIC values indicate a better model fit. Significant effects are highlighted in **bold**, trend towards significance in *italics*. Results underlined are from LME ran without the outlier identified on the scatterplots (single section in a male subject).
^1^95%CI obtained via bootstrapping.

**Supplemental Figure 1:** Flow chart detailing participant inclusion.


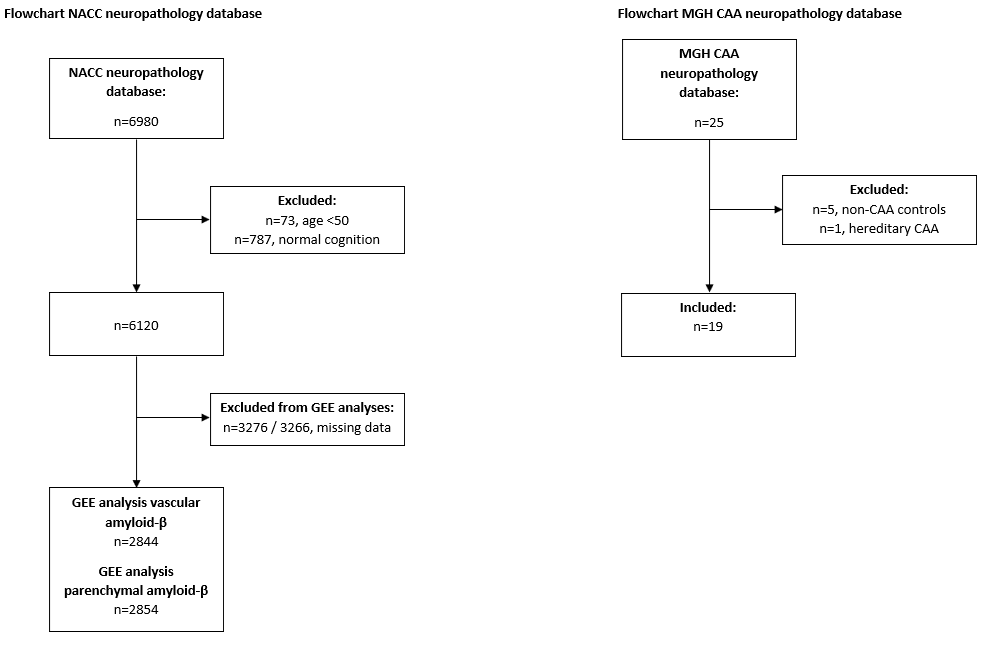


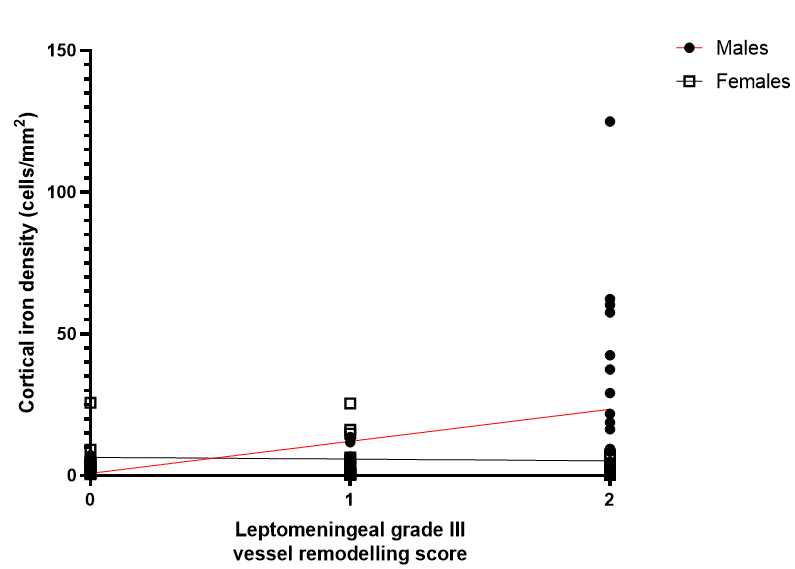

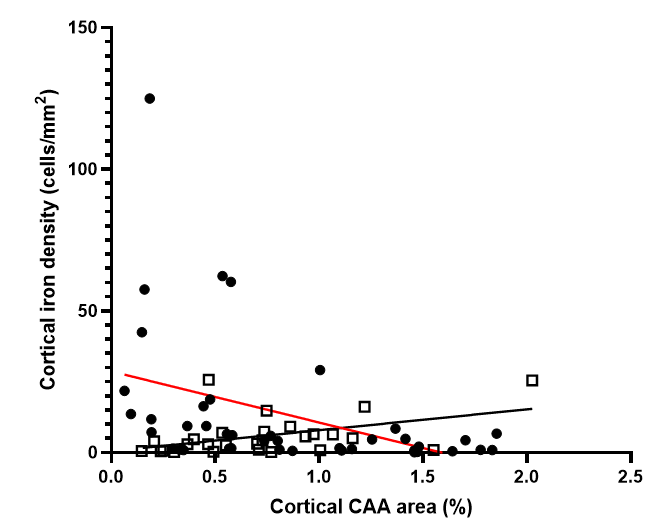
**Supplemental Figure 2**: Scatterplots illustrating cortical iron density against leptomeningeal grade III vessel remodelling score and against cortical CAA area percentage in men and women.

**B**

**A**

*Scatterplots of n=19 cases (71 sections) illustrating: (****A****) the relation between cortical iron density and cortical CAA area in men (black circles) and women (hollow squares) with simple linear regression lines to estimate direction (men red line, women black line). (****B****) the relation between cortical iron density and leptomeningeal grade III vessel remodelling score in men (black circles) and women (hollow squares), with simple linear regression lines to estimate direction (men black line, women red line). In both scatterplots the (same) outlier can be seen, a single brain section in a man (grey arrows). After exclusion of this outlier all previously found significant associations remained stable, increasing in significance (Supplemental Table 1). A stronger association of leptomeningeal grade III vessel remodelling with cortical iron density compared to women was observed after exclusion of the outlier (Supplemental Table 1).*

**References**

1. Besser L, Kukull W, Knopman DS, Chui H, Galasko D, Weintraub S, et al. Version 3 of the National Alzheimer’s Coordinating Center’s Uniform Data Set. Alzheimer Disease & Associated Disorders. 2018;32(4):351-8.

2. Vonsattel JP, Myers RH, Hedley-Whyte ET, Ropper AH, Bird ED, Richardson EP, Jr. Cerebral amyloid angiopathy without and with cerebral hemorrhages: a comparative histological study. Annals of neurology. 1991;30(5):637-49.

3. Olichney JM, Hansen LA, Lee JH, Hofstetter CR, Katzman R, Thal LJ. Relationship between severe amyloid angiopathy, apolipoprotein E genotype, and vascular lesions in Alzheimer's disease. Ann N Y Acad Sci. 2000;903:138-43.

4. Thal DR, Rüb U, Orantes M, Braak H. Phases of A beta-deposition in the human brain and its relevance for the development of AD. Neurology. 2002;58(12):1791-800.

5. Kovacs GG, Gelpi E. Clinical neuropathology practice news 3-2012: the "ABC" in AD-revised and updated guideline for the neuropathologic assessment of Alzheimer's disease. Clin Neuropathol. 2012;31(3):116-8.

6. Kozberg MG, Yi I, Freeze WM, Auger CA, Scherlek AA, Greenberg SM, et al. Blood–brain barrier leakage and perivascular inflammation in cerebral amyloid angiopathy. Brain Communications. 2022;4(5).

7. Charidimou A, Perosa V, Frosch MP, Scherlek AA, Greenberg SM, van Veluw SJ. Neuropathological correlates of cortical superficial siderosis in cerebral amyloid angiopathy. Brain : a journal of neurology. 2020;143(11):3343-51.

8. van Veluw SJ, Scherlek AA, Freeze WM, Ter Telgte A, van der Kouwe AJ, Bacskai BJ, et al. Different microvascular alterations underlie microbleeds and microinfarcts. Annals of neurology. 2019;86(2):279-92.

9. Wardlaw JM, Smith EE, Biessels GJ, Cordonnier C, Fazekas F, Frayne R, et al. Neuroimaging standards for research into small vessel disease and its contribution to ageing and neurodegeneration. The Lancet Neurology. 2013;12(8):822-38.

10. Perosa V, Oltmer J, Munting LP, Freeze WM, Auger CA, Scherlek AA, et al. Perivascular space dilation is associated with vascular amyloid-β accumulation in the overlying cortex. Acta neuropathologica. 2022;143(3):331-48.

11. Freeze WM, Bacskai BJ, Frosch MP, Jacobs HIL, Backes WH, Greenberg SM, et al. Blood-Brain Barrier Leakage and Microvascular Lesions in Cerebral Amyloid Angiopathy. Stroke. 2019;50(2):328-35.
